# Vortex beam nanofocusing and optical skyrmion generation via hyperbolic metamaterials

**DOI:** 10.1515/nanoph-2025-0315

**Published:** 2025-09-18

**Authors:** Wenhao Li, Jacob LaMountain, Evan Simmons, Jiaren Tan, Hooman Barati Sedeh, Anthony Clabeau, Robel Y. Bekele, Jason D. Myers, Takashige Omatsu, Jesse Frantz, Viktor A. Podolskiy, Natalia M. Litchinitser

**Affiliations:** Department of Electrical and Computer Engineering, 3065 Duke University, Durham, NC, USA; Department of Physics and Applied Physics, University of Massachusetts Lowell, Lowell, MA, USA; US Naval Research Lab, Washington, DC, USA; Molecular Chirality Research Center, Chiba University, Chiba, Japan; Department of Electrophysics, National Yang Ming Chiao Tung University, Taiwan

**Keywords:** hyperbolic metamaterial, vortex beams, optical skyrmion

## Abstract

While spin angular momentum is limited to ±*ℏ*, orbital angular momentum (OAM) is, in principle, unbounded, enabling tailored optical transition rules in quantum systems. However, the large optical size of vortex beams hinders their coupling to nanoscale platforms such as quantum emitters. To address this challenge, we experimentally demonstrate the subdiffraction focusing of an OAM-carrying beam using a hypergrating, a flat meta-structure based on a multilayered hyperbolic composite. We show that our structure generates and guides high-wave vector modes to a deeply subwavelength spot and experimentally demonstrate the focus of an OAM-carrying beam on a spot size of ∼ *λ*/3. We also show how the proposed platform facilitates the formation of an optical skyrmion with spin textures as small as *λ*/250, opening new avenues for controlling light–matter interactions.

## 1 Introduction

The realization that light can carry OAM, in addition to its intrinsic spin, has expanded the landscape of optical physics by introducing new degrees of freedom for encoding information, manipulating matter, and tailoring fundamental light–matter interactions [1], [2], [3]. Unlike spin angular momentum, which is limited to ±ℏ, OAM is, in principle, unbounded and associated with helical phase fronts carrying quantized topological charge, allowing controlled delivery of angular momentum to micro- and nanoscale systems, enabling a range of phenomena, including rotational Doppler shifts, angular uncertainty relations, and spin–orbit coupling, while also opening pathways to modify selection rules in quantum systems [4]. Consequently, vortex beams, structured light fields carrying OAM, have become indispensable tools in fields such as quantum information processing [5], optical micromanipulation [6], quantum communication [7], and quantum memories [8], spurring efforts to develop compact devices for generating and manipulating OAM beams on-chip [9], [10], [11], [12], [13].

However, the spatial extent of vortex beams, which scale with topological charge, inherently limits their interaction with nanoscale systems, where strong coupling requires tight spatial confinement. While structured light can be readily engineered at the macroscale, applications such as probing dipole-forbidden transitions, subwavelength imaging, and on-chip integrated optics demand significantly reduced beam sizes [14], [15], [16], [17]. To address this demand, several theoretical strategies have proposed reshaping the emitter’s photonic environment using nanostructures or metamaterials to enhance light–matter interactions [18], [19], [20], [21], [22], [23], [24], [25] locally. However, these methods often suffer from significant fabrication challenges, limited scalability, or material-related optical losses, restricting their practical use. At the same time, a growing body of theoretical work in solid-state physics and quantum optics has highlighted the potential of tightly confined vortex beams themselves, independent of emitter-side structuring, to drive new physical phenomena, including OAM-induced currents, spin-controlled magnetic responses, and transitions that are otherwise forbidden for plane waves in systems such as quantum dots, nanorings, and semiconductor heterostructures [26], [27], [28], [29], [30]. Notably, many such processes exhibit transition rates that increase as the beam size decreases [31], reinforcing the need for an optical method to compress OAM beams while preserving their topological structure. However, despite decades of theoretical developments and partial solutions, an experimental platform capable of delivering this functionality in a planar, scalable, and material-efficient form has yet to emerge.

To bridge this long-standing gap, we experimentally demonstrate a new approach for compressing vortex beams to deep subwavelength dimensions based on a flat hyperbolic metamaterial (HMM) engineered with extreme dielectric anisotropy and governed by a hyperbolic dispersion relation. The strong focusing capability of the proposed structure, referred to as a hypergrating, arises from the combination of a Fresnel grating, which generates high in-plane wavevector modes, with planar slabs of an anisotropic medium that guide and converge these modes at a focal spot [32]. Experimental measurements show that an OAM-carrying beam is focused on a spot approximately one-third of the vacuum wavelength *λ*, smaller than, the diffraction-limited spot size achieved with high-NA objective lenses [33]. We show that the measured intensity and polarization profiles are in excellent agreement with theoretical predictions, confirming the robustness of the focusing mechanism. Numerical analysis of the electromagnetic field distribution at the focal plane further reveals that, under appropriate design conditions, the structure can support the formation of optical skyrmions, characterized by spin textures as small as *λ*/250, arising from the interplay between spin–orbit coupling and tightly confined optical modes.

## 2 Concept and theoretical prediction

**Figure 1:**
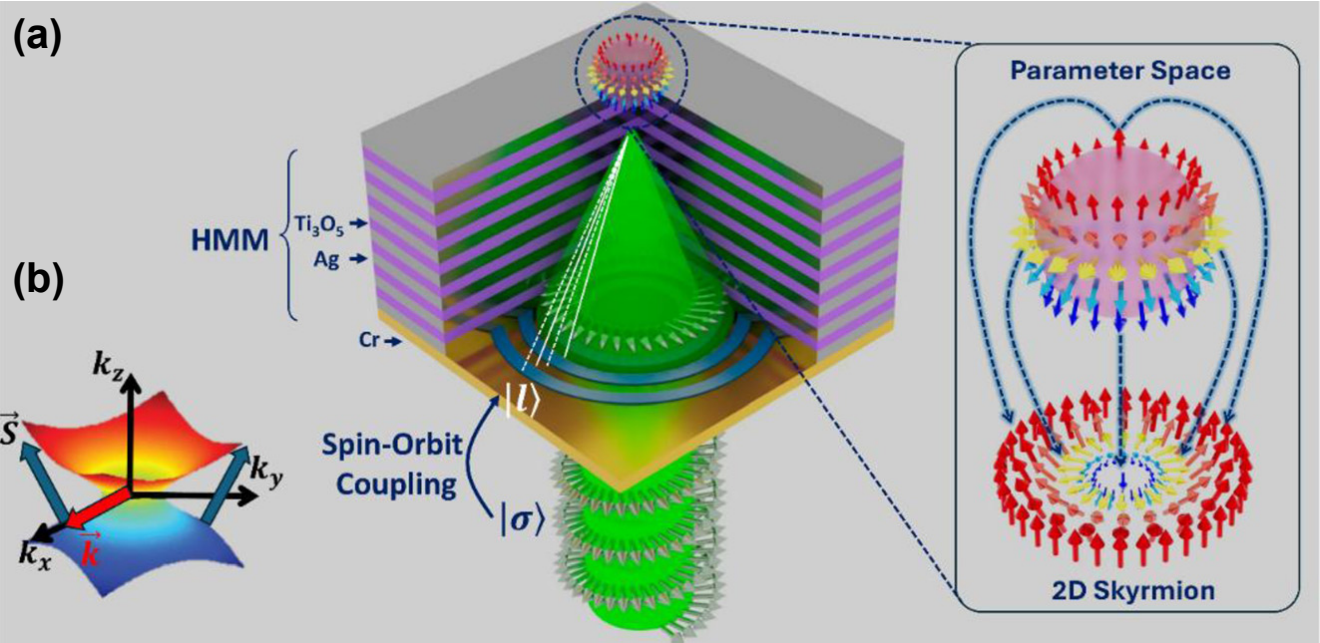
Schematic diagram of a hypergrating structure and its working principle. (a) The Fresnel grating ensures that the beams leaving the slits in the direction of the focal spot interfere constructively with each other. Solid and dashed white lines represent directions of the interfering beams (as defined by their Poynting flux); phase shifts between the beams propagating along two solid lines and two dashed lines are multiples of 2π, while the phase shift between the beams propagating along neighboring solid and dashed lines is π. (b) Illustration of the hyperbolic effective medium response of the metamaterial (note the negative phase velocity of the plane waves).

The internal structure of vortex beams inherently contains phase singularities, which fundamentally distinguish them from Gaussian beams and introduce challenges for tight spatial focusing, such that even under high numerical aperture focusing, beams carrying a topological charge of 1 typically produce focal spots larger than the free-space wavelength, with diameters exceeding *λ* by 10 % [33]. To tackle this problem, here, we propose a flat HMM slab that is combined with a diffractive element to form a compact focusing platform, referred to as a *hypergrating*, which simultaneously generates high in-plane wavevector components and guides them toward a deeply subwavelength focal region [32]. In particular, as shown in Figure 1a, circularly polarized light is first converted into a vortex beam, which is then coupled into and compressed within the HMM, resulting in strong spatial confinement of the beam’s angular momentum content.

The HMM is composed of a ten-bilayer Ag/Ti_3_O_5_ stack designed to operate in the type-II hyperbolic regime at wavelength of 532 nm. The dielectric response of the HMM is modeled using effective medium theory (EMT), with in-plane and out-of-plane components given by $\varepsilon_{\|}=f\varepsilon_{1}+\left(1-f\right)\varepsilon_{2}$ and $\varepsilon_{⊥}={\left[f/\varepsilon_{1}+\left(1-f\right)/\varepsilon_{2}\right]}^{-1}$ , where *ɛ*_1_ = 5.13 and *ɛ*_2_ = −11.5571 + 0.3672*i* are the permittivities of Ti_3_O_5_ and Ag, respectively, and *f* = 0.5 is the filling factor. These values yield *ɛ*_‖_ = −3.2135 + 0.1836*i* and *ɛ*_⊥_ = 18.4227 + 0.4664*i*, confirming the realization of a strongly anisotropic medium exhibiting type-II hyperbolic dispersion [34], [35], (see Figure 1b) demonstrating the presence of extremely large in-plane wavevectors necessary for mediating the high spatial confinement observed in the focused OAM beam (see Supplementary Material for details on the experimental characterization of the planar HMM). While EMT provides a valid first approximation, its assumptions break down for multilayer structures with relatively thick individual layers (∼30 nm), where deviations from homogenized behavior become non-negligible [36]. To account for these effects and accurately determine the spatial modulation pattern, we adopted the formalism for periodically stratified media [37] to calculate the precise boundaries of the Fresnel zones across the HMM slab. In the resulting design, the odd-numbered zones are left transmissive while the even-numbered zones are blocked, enabling constructive interference of the transmitted beam at the designed focal plane, as illustrated in Figure 1a.

## 3 Nanofocusing of vortex beams

**Figure 2:**
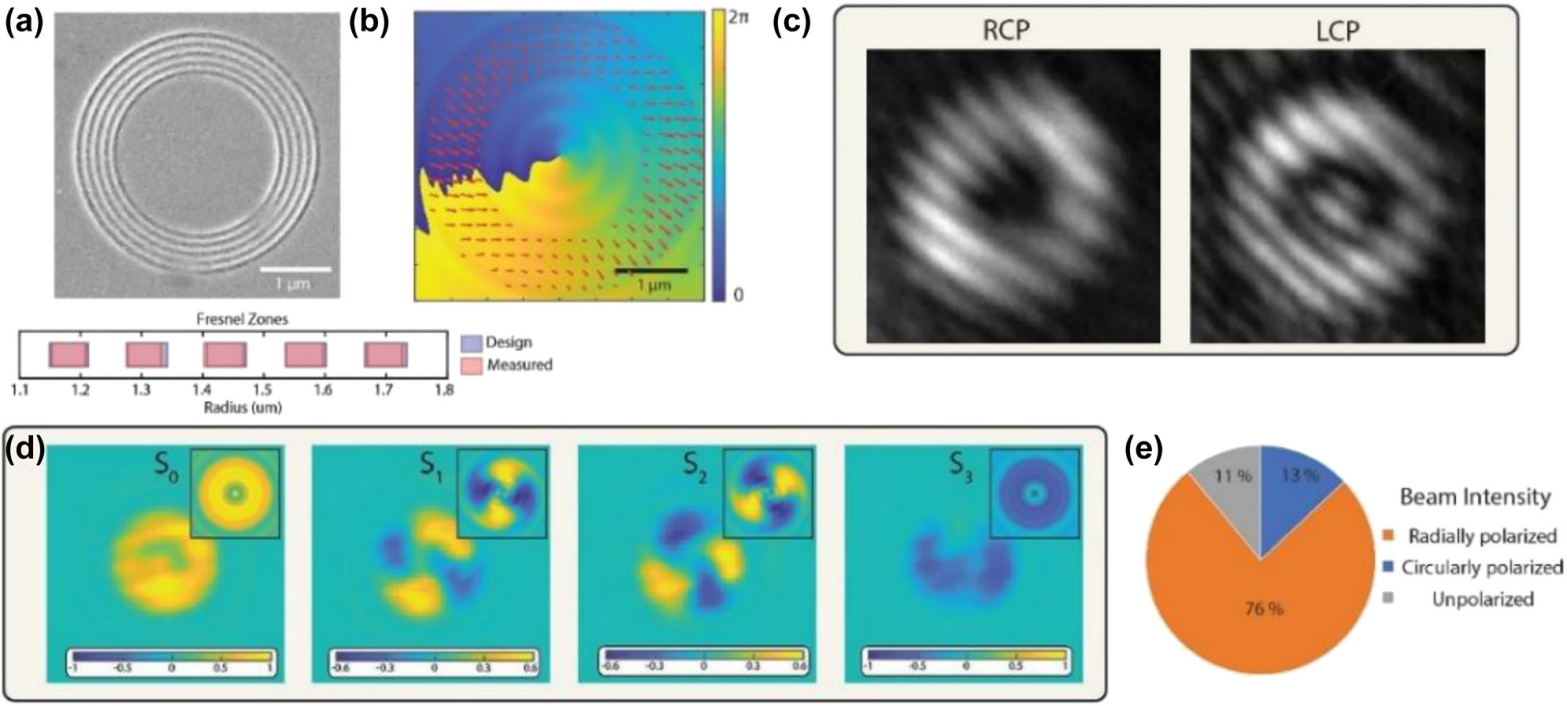
Spin- to orbital-angular momentum conversion. (a) Scanning electron microscope (SEM) image of a Fresnel grating and the Fresnel grating rings’ radii (designed and experimentally measured). (b) Vector plot of the electric field (red arrows) in the transverse plane and the phase distribution of the radially polarized component (*E*_*r*_) of the beam at 60 nm after the Fresnel grating. (c) Interference pattern of the right-handed circularly polarized (RCP) and left-handed circularly polarized (LCP) components of the output beam. (d) Experimentally measured Stokes parameters distributions in the far field (inserted: Stokes parameters calculated from simulation). (e) Intensity distribution among components of the beam after the Fresnel grating.

A Fresnel grating was fabricated by milling the odd Fresnel zones on a Cr film, as shown in Figure 2a. Owing to the subwavelength radial spacing between adjacent Fresnel zones, the grating exhibits a pronounced polarization-dependent transmission behavior, wherein radially polarized light is transmitted with significantly higher efficiency than its azimuthally polarized counterpart, such that when a circularly polarized beam is incident on the structure, the azimuthal component is preferentially absorbed or reflected, while the radial component passes through, resulting in an effective conversion of the incident spin angular momentum into orbital angular momentum. To quantify the relative contributions of OAM and residual SAM content in the output beam, we performed full-wave simulations in COMSOL by modeling the transmission of a circularly polarized beam through the Cr Fresnel grating (see Supplementary Material). As shown in Figure 2b, the electric field vectors in the transmitted beam exhibit a predominantly radial orientation, consistent with the generation of an OAM-carrying mode [38]. From the simulated field distribution, we estimate that approximately 83 % of the transmitted power corresponds to the radially polarized OAM beam, with the remaining 17 % associated with residual circular polarization. The SAM to OAM conversion was confirmed by acquiring the phase information of the output beam using an interference measurement. While direct interference measurement of a polarized beam is tricky, the radially polarized beam with topological charge *l* = −1 can be represented as a linear combination of left-handed and right-handed circularly polarized beams with charge *l* = 0 and *l* = −2 as [39]:

(1) $$\left[\begin{array}{c} \cos\left(\varphi \right)\\ \sin\left(\varphi \right) \end{array}\right]e^{-i\varphi }=\frac{1}{2}\left[\begin{array}{c} 1\\ -i \end{array}\right]+\frac{1}{2}\left[\begin{array}{c} 1\\ +i \end{array}\right]e^{-2i\varphi }$$

where *φ* denotes the azimuthal angle.

**Figure 3:**
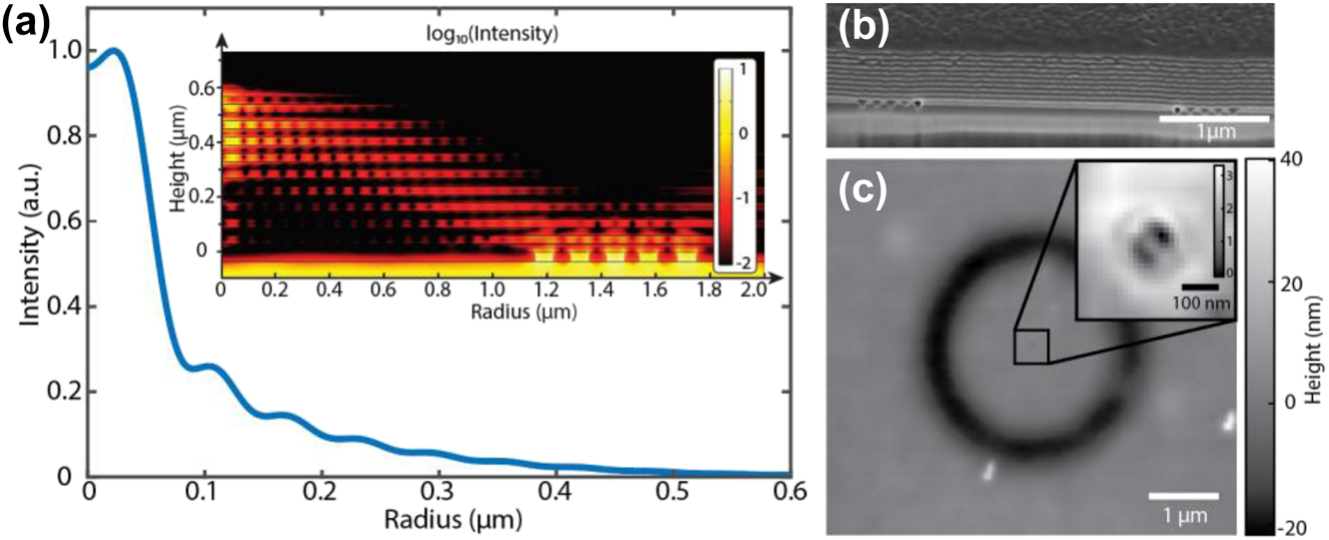
Generation and nano-focusing of OAM beam. (a) Light intensity distribution at the focus of the hypergrating (10 nm above the HMM) from numerical simulation. The inserted figure shows the light intensity distribution in HMM. Note that the intensity distribution has axial symmetry with respect to the principal axis. (b) Cross section of the hypergrating structure. (c) The surface topology of the Azo-polymer after the exposure measured by atomic force microscopy.

The total topological charges associated with the left-handed and right-handed circularly polarized components of the transmitted beam were measured to be 0 and –2, respectively, as shown in Figure 2c, from which it can be inferred (based on the angular momentum decomposition) that the output field contains a dominant OAM component with topological charge −1, corresponding to the expected vortex mode generated by the polarization-selective transmission through the Fresnel grating. To experimentally quantify the contribution of the radially polarized OAM beam in the transmitted field, the polarization state of the output beam was measured using the method described in [40], [41] and detailed further in the Supplementary Material, with the resulting spatial distribution of Stokes parameters shown in Figure 2d. In particular, it was determined that the radially polarized OAM component accounts for approximately 76 ± 1 % of the total transmitted intensity, while an additional 11 % of the beam appears to lose its defined polarization state after transmission through the Fresnel grating, an effect that is likely attributable to scattering-induced depolarization originating from surface roughness or fabrication imperfections (see Supplementary Material for details). Following the confirmation of OAM beam generation, the Fresnel grating was integrated onto the planar HMM to realize the complete hypergrating structure, and the subsequent propagation of light through this system was investigated both numerically and experimentally to assess the focusing behavior and field confinement enabled by the combined action of the grating and the hyperbolic medium. Figure 3a presents the numerically predicted performance of the hypergrating structure under illumination by a circularly polarized beam at a wavelength of 532 nm, where the simulated intensity distribution, evaluated 15 nm above the top surface of the HMM, reveals a tightly focused beam with a full width at half maximum (FWHM) of approximately 110 nm, corresponding to about λ/5. Notably, the same hypergrating configuration is capable of focusing vortex beams carrying higher-order OAM charges with similar subdiffraction-limited resolution, outperforming what can be achieved using conventional high-numerical-aperture (NA) objective lenses (see Supplementary Material). This enhanced focusing capability originates from the propagation of high in-plane wavevector modes that are supported by the hyperbolic dispersion of the metamaterial slab, modes that cannot exist in isotropic materials such as air or immersion oil, and thus facilitate energy compression well below the free space diffraction limit. It is important to note that, due to total internal reflection at the top interface, the focused beams are not directly outcoupled into free space but instead form an evanescent field localized just above the HMM surface.

A cross-sectional scanning electron microscope (SEM) image of the fabricated hypergrating structure is shown in Figure 3b, and further details of the fabrication procedure are provided in the Supplementary Materials. To experimentally characterize the near-field intensity distribution at the focal plane of the hypergrating, we employed an Azo-polymer film specifically designed for sensitivity to 532 nm illumination, which undergoes photoinduced isomerization between its *Trans* and *Cis* states, thereby forming nanoscale surface relief structures in response to light exposure [42]. As shown in Figure 3c, following a 5-min exposure to a 532 nm continuous-wave laser at an intensity of *I*_inc_ = 216 W/cm^2^, the Azo-polymer revealed a distinct figure-eight-shaped surface modulation pattern located at the expected focal region of the hypergrating. This structure appears superimposed on a preexisting, donut-shaped valley approximately 20 nm deep (see Supplementary Materials) and is attributed to the interaction of the tightly focused OAM beam with the polymer surface. The relief feature at the focal point has a lateral dimension of approximately 200 nm and a peak-to-valley height of ∼3 nm, corresponding to an estimated optical spot size of *λ*/2.7, which, while slightly larger than the predicted *λ*/5, still clearly demonstrates subdiffraction focusing. It is noteworthy to mention that the surface relief pattern does not map directly onto the light intensity distribution due to the complex photomechanical response of the Azo-polymer and the presence of both transverse and longitudinal electric field components at the focal point, as discussed in prior work [43] and detailed in the Supplementary Materials. In other words, the observed figure-eight-shaped surface relief pattern is not a direct mapping of the intensity profile but rather arises from photoinduced mass migration driven by interference between the longitudinal and transverse field components near the surface, similar to mechanisms discussed in Ref. [43]. This interference becomes allowed due to symmetry breaking at the polymer–air interface and leads to surface deformations that encode the spin and phase structure of the focused field. Deviations from theoretical beam size may also arise from fabrication-induced imperfections, such as surface roughness and slight nonplanarity in the multilayer stack. We note that the ability of the hypergrating to tightly localize OAM beams holds important implications for enhancing light–matter interactions at the nanoscale. Prior theoretical studies based on multipolar expansions of electric and magnetic fields have shown that vortex beams tightly confined to subwavelength scales can give rise to new types of optical transitions in quantum emitters, particularly in quantum dots, that are otherwise forbidden for plane waves at normal incidence [14], [29]. Moreover, unlike conventional vortex beams, in which the intensity minimum near the singularity complicates alignment with atomic systems, the hypergrating enables the generation of a high-intensity focal spot, thus facilitating precise spatial overlap between the vortex field and nanoscale targets [44], [45]. We also note that the proposed framework is readily extendable to higher-order modes, offering a scalable platform for nanoscale manipulation of structured light (see Supplementary Materials).

## 4 Optical quasiparticles

Originally introduced by Tony Skyrme in 1961 as topologically protected quasiparticles describing solitonic structures in nucleons, skyrmions have since emerged in a variety of physical systems, including magnetic materials, liquid crystals, Bose–Einstein condensates, and, more recently, optical fields [46]. In optics, skyrmions are realized as structured vector fields characterized by nontrivial mappings of polarization or spin textures onto the unit sphere, with their topology defined by their skyrmion numbers. The hypergrating platform we developed for subdiffraction focusing of OAM beams inherently produces a tightly confined and spatially structured electromagnetic field at the focal region, where strong spin–orbit coupling coexist.

**Figure 4:**
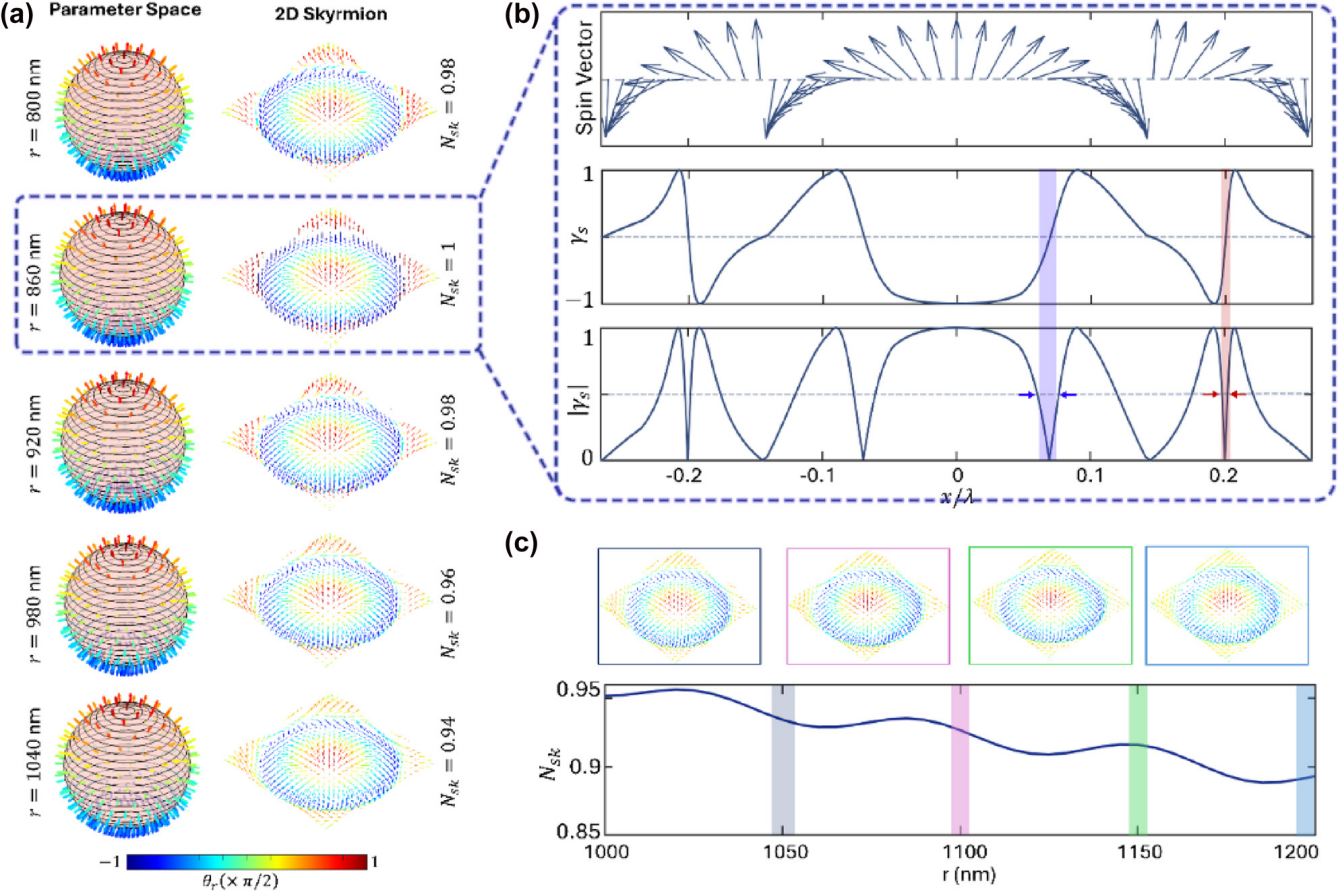
Analysis of spin-vector distribution in the vicinity of focal spot. (a) Normalized spin vector fields σ plotted at 15 nm above the HMM surface for various Fresnel grating radii, showing the evolution of spin textures as the radius increases from 860 nm to 1,040 nm. Hedgehog-like textures characteristic of Néel-type skyrmions are observed at *r* = 860 nm (dashed blue line), while more complex and nontopological textures emerge for larger radii. The skyrmion number *N*_*sk*_ is computed for each configuration, showing a quantized value of *N*_*sk*_ = 1 for the 860 nm case and a gradual decrease (∼0.02 per step) for larger radii. (b) Top row: cross-sectional profiles of the spin vector **σ** for *N*_*sk*_ = 1, illustrating the radial spin reversal near the beam center and a second reversal in the outer domain. Bottom rows: local spin structure parameter $\gamma_{s}=\left(I_{\text{RCP}}-I_{\text{LCP}}\right)/\left(I_{\text{RCP}}+I_{\text{LCP}}\right)$ visualized for the 860 nm configuration, demonstrating deep-subwavelength spin-flip transitions of 8 nm $\left(\approx \lambda /67\right)$ and 2 nm ( ≈λ/250 ) within the first and second spin domains, respectively, as highlighted by blue and red shaded colors. (c) Spin vector fields for four additional Fresnel grating radii of 1050 nm (dark blue), 1,100 nm (magenta), 1,150 nm (green), and 1,200 nm (light blue) are shown as representative examples of increasingly complex but nonquantized spin textures.

As a result, the same structure can serve not only as a tool for deep-subwavelength beam compression but also as a compact and planar source of optical skyrmions, enabling experimental access to topologically nontrivial light fields that hold potential for applications in nanoscale imaging, ultrafast vectorial field mapping, and topological photonic information processing [46]. To demonstrate the capability of the proposed hypergrating in generating optical skyrmions, we performed a parametric study by varying the radius of the Fresnel grating from 800 nm to 1,040 nm and evaluated the resulting electric and magnetic field distributions at a plane located 15 nm above the HMM surface (i.e., approximately 615 nm from the source plane), where the focal region forms and the vectorial field structure becomes strongly pronounced. We analyzed the vectorial spin texture formed in the focal region by computing the spin angular momentum density, defined as $S=\text{Im}\left(\varepsilon \;E^{*}\times E+\mu \;H^{*}\times H\right)/4\omega$ , where **E** and **H** are the electric and magnetic fields, *ɛ* and *μ* denote the free-space permittivity and permeability, respectively, and *ω* is the optical frequency. The resulting SAM field was then normalized as $\sigma =S/\left|S\right|$ , to provide a direct and spatially resolved measure of the local spin structure in the focal plane as shown in Figure 4a. As evident from this panel, increasing the radius of the Fresnel grating leads to progressive modifications in the field distributions within the hypergrating, such that increasingly complex spin textures begin to emerge and continuously evolve into distinct configurations as the radius increases further, with the color of each arrow indicating the direction of the unit spin vector σ, determined by the angle $\theta_{r}=\tan^{-1}\left(S_{z}/S_{r}\right)$ [47]. Although the spin textures associated with different Fresnel grating radii may appear qualitatively similar, both in their representation on the parameter sphere and in their projected trajectories onto the unit circle, the corresponding skyrmion numbers provide a more precise and quantitative measure of their topological distinction [47]. In particular, the topology of a skyrmionic configuration can be characterized by the skyrmion number defined as

(2) $$N_{sk}=\frac{1}{4\pi }\iint S\cdot \left(\frac{\partial S}{\partial x}\times \frac{\partial S}{\partial y}\right)\text{d}x\text{d}y$$

where the integration is carried out over the region confining the topological quasiparticle. Upon evaluating this integral across different textures, we find that the skyrmion number decreases gradually by approximately 0.02 between successive configurations, suggesting structural similarities among the spin fields; however, this smooth variation does not imply topological equivalence, as even small deviations in the skyrmion number reflect nontrivial changes in the vector field topology.

Most notably, for the specific case of *r* = 860 nm, the skyrmion number is found to be exactly *N*_*sk*_ = 1, which unambiguously confirms the presence of a topologically protected Néel-type skyrmion, characterized by its hedgehog-like spin texture. This discrete quantization at unity serves as a clear topological marker that distinguishes this configuration from neighboring cases, whose skyrmion numbers deviate from integer values and, therefore, do not correspond to classical skyrmionic quasiparticles. For the specific case corresponding to the Néel-type skyrmion at *r* = 860 nm, where the skyrmion number is quantized at *N*_*sk*_ = 1, the spin vector exhibits a distinct and continuous reversal from the “up” to the “down” state along the radial direction, as shown in the top row of Figure 4b, illustrating the hedgehog-like spin topology associated with this configuration. To demonstrate that the reversal of the spin vector within the domains occurs at deep subwavelength scales, we introduce a spin-related contrast parameter $\gamma_{s}=\frac{I_{\text{RCP}}-I_{\text{LCP}}}{I_{\text{RCP}}+I_{\text{LCP}}}$ , where *I*_RCP_ and *I*_LCP_ denote the intensities of the right- and left-handed circularly polarized components of the transverse field at a given spatial point [47]. In this framework, *γ*_*s*_ = +1 (or −1) corresponds to pure right-handed (or left-handed) circular polarization, *γ*_*s*_ = 0 indicates a locally linear polarization, and fractional values reflect elliptical polarizations, thereby allowing a spatially resolved quantification of the local spin state associated with the skyrmion texture. We calculated the high-resolution cross-sectional profile of the spin state reversal, and its result is shown in the second row of Figure 4b, revealing a rapid transition of the local spin from positive to negative values over deeply confined spatial regions (blue shaded box), highlighting the strong vectorial field modulation near the skyrmion core. In addition to the primary spin reversal near the beam center, a second spin vector reversal is also observed further from the core, as marked by the light red shaded area in Figure 4b, further emphasizing the complex multidomain nature of the skyrmion-like texture supported by the hypergrating. In particular, the spin-flip occurring over remarkably short spatial scales of 8 nm ( ≈λ/67 ) and 2 nm ( ≈λ/250 ) within the first and second domains, as shown in the third row of Figure 4b, highlights the deeply subwavelength nature of the topological transition. We note that compared to the deep-subwavelength photonic skyrmions reported in [47], where the second spin-vector reversal (from the beam center) occurs over ≈10nm on a single interface, our hypergrating platform achieves a much tighter spin-texture modulation of ≈2 nm, corresponding to *λ*/250 at 532 nm, which stems from the carefully designed type-II hyperbolic metamaterial in our work. To further assess the nature of spin textures beyond this quantized case, we also plotted the spin vector fields for the larger grating radii of 1,000 nm and 1,200 nm, where the skyrmion number deviates from unity. As shown in Figure 4c, the resulting spin distributions, while still exhibiting spatial complexity and nontrivial structure, lack the clear hedgehog-like topology characteristic of the Néel-type configuration, indicating that although these textures are rich in vectorial content, they do not meet the topological criteria associated with classical optical skyrmions. It should also be remarked that due to the rotational symmetry of the hypergrating, light propagation is best described using orthogonal radial and azimuthal polarization bases. In this perspective, circularly polarized light decomposes into radially and azimuthally polarized components, corresponding to transverse electric (TE) and transverse magnetic (TM) waves in the HMM, respectively. While TM waves enable subdiffraction focusing, TE modes exhibit dispersion nearly identical to free space. Within the HMM, TM waves naturally decouple from TE components during propagation and become focused by the hypergrating. Consequently, residual spin angular momentum does not disrupt skyrmion textures. We note that although direct experimental observation of the optical skyrmion was not performed in this work, the use of near-field scanning optical spectroscopy could, in future studies, enable the spatial resolution of both the skyrmionic spin structure and the rapid spin-flip transitions occurring at deep subwavelength scales, thereby providing further experimental validation of the topological textures supported by the hypergrating [48].

## 5 Conclusions

In summary, we proposed and experimentally demonstrated a planar hypergrating platform capable of sub-diffraction focusing of vortex beams and supporting the formation of optical skyrmions. Numerical simulations predict that the radially polarized OAM beam can be focused down to *λ*/5 due to the excitation of high in-plane wavevector modes within the hyperbolic metamaterial, achieving spatial confinement far beyond that of high-NA objective lenses. The spin texture at the focal plane reveals deep-subwavelength topological structure, with spin-flip transitions occurring over distances as small as *λ*/250, consistent with the emergence of a Néel-type optical skyrmion. Experimental realization of the hypergrating confirms beam compression to approximately λ/3, validating the strong focusing capability of the platform. Beyond enhanced field confinement, the demonstrated system provides a compact and versatile route toward topological field engineering and offers new opportunities for controlling light–matter interactions at the nanoscale, particularly in the context of chiral quantum systems and OAM-based photonic applications.

## Supplementary Material

Supplementary Material Details
