# Supplementary material for: Vortex beam nanofocusing and optical skyrmion generation via hyperbolic metamaterials
Source: Nanophotonics. 2025 Sep 18;14(25):4545–53. doi: 10.1515/nanoph-2025-0315 (PMC12714047; doi:10.1515/nanoph-2025-0315)
Supplement: Supplementary file 1 — Supplementary Material Details [file j_nanoph-2025-0315_suppl_001.pdf]

Supplementary Material
Vortex Beam Nanofocusing and Optical Skyrmion Generation via Hyperbolic Metamaterials

Wenhao Li,[1] Jacob LaMountain,[2] Evan Simmons,[2] Jiaren Tan,[1] Hooman Barati Sedeh,[1] Anthony Clabeau,[3] Robel Y. Bekele,[3] Jason D. Myers,[3] Takashige Omatsu,[4] Jesse Frantz,[3] Viktor A. Podolskiy,[2,] and Natalia M. Litchinitser[1,*]

[1] *Department of Electrical and Computer Engineering, Duke University, Durham, NC, USA*
[2] *Department of Physics and Applied Physics, University of Massachusetts Lowell, Lowell, MA, USA*
[3] *US Naval Research Lab, Washington, DC, USA*
[4] *Molecular Chirality Research Center, Chiba University, Chiba, Japan*
[*] e-mail: natalia.litchinitser@duke.edu

S1. Optical Response of Planar HMM

The optical response of the planar hybrid hyperbolic metamaterial (HMM) was studied using spectroscopic ellipsometry measurements. The change in polarization state upon reflection is represented by amplitude ratio $\tan(\Psi)$ and phase difference Δ: $\tan(\Psi)\, e^{i\Delta} = \frac{\widetilde{r_p}}{\widetilde{r_s}}$, where $\widetilde{r_p}$ and $\widetilde{r_s}$ are the Fresnel reflection coefficients for the p- and s-polarized light, respectively. Ψ and Δ were analyzed by measuring at incident angles of 65°, 70°, and 75°. To determine the thicknesses of the Ag and Ti_3O_5 layers, the measured Ψ and Δ curves were fitted to the model predicted using the transfer matrix method, given the refractive indices of the individual Ag and Ti_3O_5 layers.

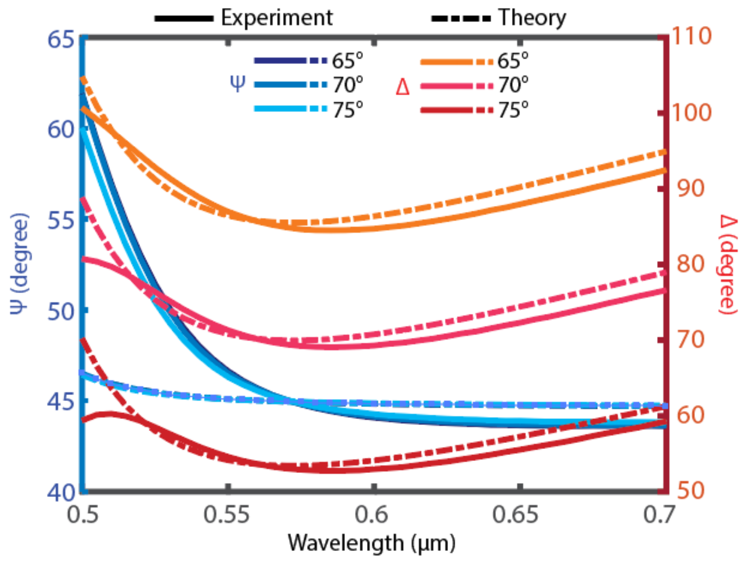

FIG. S1. Ellipsometry measurement of the 10-bilayer HMM in reflection mode.

Figure S1 depicts the experimentally measured Ψ and Δ curve as a function of wavelength fitted to a 10-bilayer model with the thickness of Ag and Ti_3O_5 as fitting parameters. The fitting model for Ψ and Δ assumes identical thicknesses for all Ag and Ti_3O_5 layers. The determined thickness values from the fitting process are as follows: Ag layers have a thickness of 28.8 nm, and Ti_3O_5 layers have a thickness of 32.0 nm.

S2. Numerical Simulations

Numerical simulations were conducted using COMSOL Multiphysics to model the electromagnetic response of the hypergrating structure. The simulation domain exploited axial symmetry, and the excitation source was a circularly polarized plane wave at a vacuum wavelength of 532 nm. The hypergrating design was implemented on a glass substrate and comprised a 50 nm thick chromium Fresnel grating layer overlaid with 20 alternating layers of Ag and Ti_3O_5, each 30 nm thick. The material refractive indices were experimentally obtained via ellipsometry and

incorporated into the simulation model. The spatial locations of the Fresnel zones were determined using the effective medium dispersion relation of the hyperbolic metamaterial HMM, as detailed in the following section.
To evaluate the spin angular momentum distribution and characterize the skyrmionic textures, the simulated electromagnetic fields were extracted from the axially symmetric model and transformed into Cartesian coordinates using COMSOL's LiveLink for MATLAB. The SAM was then calculated using the analytical expressions provided in the main text. The parameter space mapping (Poincaré-like sphere) and the two-dimensional skyrmion textures were visualized using a custom-developed MATLAB function. From simulations accounting for material absorption, the electric field amplitude at the HMM–air interface was found to be approximately 14% of that at the hypergrating input plane.

S3. Fresnel Grating Design

To realize sub-diffraction focusing using the high in-plane wavenumber modes, we engineered the light propagation to realize constructive interference at the output plane (corresponding to the focal point) of the hypergrating. The input plane of the hypergrating was divided into concentric rings based on the phase acquired by the light during its propagation from the input plane to the focal point. However, the light propagation in the HMM is not well described by the effective medium theory when the layers in the HMM are relatively thick (in our case, they are 30 nm). Therefore, we describe modes supported by HMM using expressions developed for periodically stratified media [1]. The dispersion of modes (the dependence of the components of the wavevector k_x, k_z on the angular frequency ω) is given by

$$f(k_x, k_z, \omega) = \cos(2k_z a) - \cos(k_1 a)\cos(k_2 a) + \gamma \sin(k_1 a)\sin(k_2 a) = 0, \quad \text{(S1)}$$

where a is the layer thickness, γ is a polarization-specific parameter given by

$$\gamma_{\mathrm{TM}} = \frac{1}{2}\left(\frac{\epsilon_2 k_1}{\epsilon_1 k_2} + \frac{\epsilon_1 k_2}{\epsilon_2 k_1}\right), \qquad \gamma_{\mathrm{TE}} = \frac{1}{2}\left(\frac{k_1}{k_2} + \frac{k_2}{k_1}\right), \qquad k_{1,2}^2 = \epsilon_{1,2}\left(\frac{\omega}{c}\right)^2 - k_x^2 \quad \text{(S2)}$$

The direction of the pulse propagating in the layered media can be calculated by analyzing the Poynting vector. In the lossless limit,

$$S_x \propto \frac{\partial \omega}{\partial k_x} = \frac{\partial f}{\partial k_x}\left(\frac{\partial f}{\partial \omega}\right)^{-1}, \ S_z \propto \frac{\partial \omega}{\partial k_z} = \frac{\partial f}{\partial k_z}\left(\frac{\partial f}{\partial \omega}\right)^{-1} \quad \text{(S3)}$$

The design of the grating employs the above relationship between the direction of the wavevector (phase velocity) and that of the beam propagation (group velocity), along with the requirement for the beams from the neighboring Fresnel zones to arrive at the focal point with a phase shift of π. Assuming the normally-incident plane wave field at the grating and the target focal length f_0, the edges of the Fresnel zones are given by:

$$\left(k_{x_i} x_i + k_{z_i} f_0\right) - \left(k_{x_0} x_0 + k_{z_0} f_0\right) = m\pi, \quad \text{(S4)}$$

where x_{j} is the position of the j-th Fresnel zone edge and $\left\{k_{x_j}, k_{z_j}\right\}$ represent the components of the wavevector of the beam propagating from the edge of the zone to the focal point. Note that for type II HMMs, used in this work, x_0 needs to be larger than the cutoff range, which is calculated to be 1.07 µm in the lossless approximation. The optimization of the x_0 position is performed with simulations. The best focusing is achieved at $x_0 = 1.10$ µm in the lossless approximation and $x_0 = 0.88$ µm with losses. Experimentally, the best focusing was achieved for $x_0 = 1.15$ µm. The difference in the experiment and the theory might be due to the roughness-induced scattering or inaccuracies in the film thickness and refractive indices.

S4. Spin Angular Momentum to Orbital Angular Momentum Conversion

Circularly polarized beams can be decomposed into a combination of radially polarized and azimuthally polarized components with a spiral phase wavefront. Here, the period of the gratings is much smaller than the incident beam wavelength, and the transmitted beam is predominantly radially polarized if the azimuthal component of the field is strongly absorbed or reflected.

$$\begin{gathered}\vec{E}_{\mathrm{in}} = E_0\left(\hat{e}_{\mathrm{x}} - i\hat{e}_y\right) = E_0 e^{-i\varphi}\left(\hat{e}_r - i\hat{e}_\varphi\right), \\ \vec{E}_{\mathrm{out}} = \alpha E_0 e^{-i\varphi}\hat{e}_r + (1-\alpha)(E_0 e^{-i\varphi}\hat{e}_r - iE_0 e^{-i\varphi}\hat{e}_\varphi)\end{gathered} \quad \text{(S5)}$$

where α is the fraction of the circularly polarized light that is incident onto the rings of the grating and converted to the radial polarization, and $(1-\alpha)$ is the fraction of the circularly polarized input beam that was not converted due to imperfect absorption and reflection of the azimuthally polarized component by the rings. The output beam becomes a predominantly radially polarized vortex beam due to the conversion of the spin angular momentum to orbital angular momentum.

S5. Stokes parameters analysis

The Stokes parameters measurement was based on four intensity measurements with the setup shown in FIG. S2, and is known as reduced Stokes polarimetry[2]. The output beam was collected with an objective (Nikon, 100x, 0.8NA), passed through the quarter-wave plate (QWP) and a linear polarizer (LP), and then recorded with a camera (Princeton Instrument, Pixis). The circular intensity profiles (I_R and I_L) were acquired by setting the QWP at 90 degrees and the LP at 45 and 135 degrees, respectively. By removing the QWP and adjusting the polarizer to angular orientations of 0 and 45 degrees, the two linear intensities (I_D and I_H) were acquired. The Stokes parameters were obtained using the following equations: $S_0 = I_R + I_L$, $S_1 = 2I_H - S_0$, $S_2 = 2I_D - S_0$, $S_3 = I_R - I_L$.

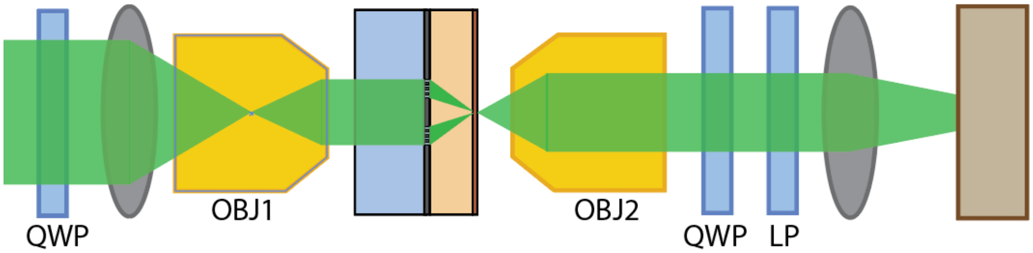

FIG. S2. Experimental setup for measuring the Stokes parameters.

The output beam of the Fresnel grating can be represented as a linear combination of a radially polarized vortex beam and a circularly polarized beam described by Eq. (S5), which can be rewritten as

$$\vec{E}_{\text{out}} = E_{r0} e^{-i\varphi} \hat{e}_r + \frac{E_{s0}}{\sqrt{2}} (\hat{e}_x - i\hat{e}_y), \qquad \text{(S6)}$$

where $E_{r0} = \alpha E_0$ and $E_{s0} = (1-\alpha)\sqrt{2}E_0$are the electric field distributions of the vortex and circularly polarized beam components. From the generalized form of Stokes parameters [2], the output beam of the Fresnel grating has the Stokes parameters with the following form:

$$\begin{aligned} S_0 &= E_{r0}^2 + E_{s0}^2 + \sqrt{2}E_{r0}E_{s0} \\ S_1 &= \left(E_{r0}^2 + \sqrt{2}E_{r0}E_{s0}\right)\cos(2\phi) \\ S_2 &= \left(E_{r0}^2 + \sqrt{2}E_{r0}E_{s0}\right)\sin(2\phi) \\ S_3 &= E_{s0}^2 + \sqrt{2}E_{r0}E_{s0} \end{aligned} \qquad \text{(S7)}$$

S_1 and S_2 distributions have four quarters and are rotated by 45 degrees. The Stokes parameters distributions S_0, S_1, S_2, and S_3 from experimental measurement and simulation are shown in the main paper, Fig. 2(d). From S_1, S_2, and S_3, the magnitude of the E_{s0} and E_{r0} distributions can be calculated. Figure S3 (a) and (b) show the E_{s0}^2 and E_{r0}^2 distribution of a circularly polarized beam passed through the Fresnel gratings. The total intensities of the vortex beam and circularly polarized are determined using the following equations,

$$I_v = \iint E_{r0}^2 dxdy\,, \; I_s = \iint E_{s0}^2 dxdy \qquad \text{(S8)}$$

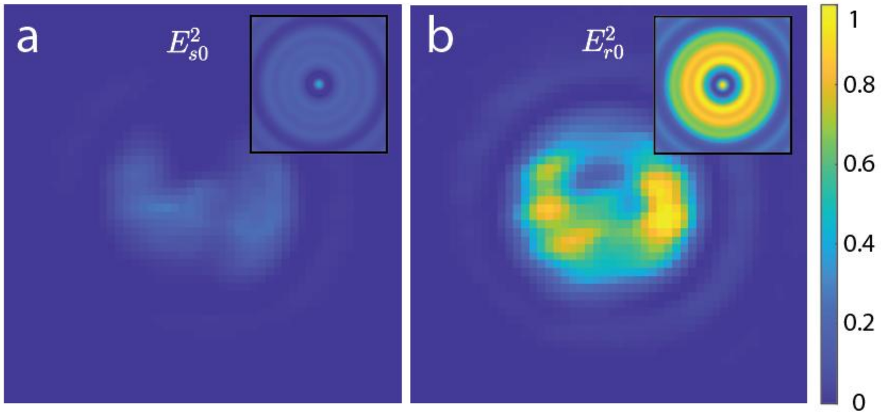

FIG. S3. E_{s0}^2 and E_{r0}^2 distributions calculated from the Stokes parameters.

In the simulation results, the circularly polarized beam accounts for 17% of the total power, while the radially polarized vortex beam constitutes the remaining 83%. In the experiment, the degree of polarization, defined as $p = \frac{I_{pol}}{I_{tot}} = \sqrt{S_1^2 + S_2^2 + S_3^2}/S_0$, is measured to be 0.89. The contributions to the total intensity from the unpolarized beam, circularly polarized beam, and radially polarized vortex beam are 11%, 13%, and 76%, respectively. The loss of polarization might be due to scattering.

S6. Focusing High-Order OAM Beam with Hypergrating

The focusing behavior of a high OAM vortex beam with the hypergrating device was studied using COMSOL simulations. The input beam is a radially polarized beam defined as $E_{in} = E_0(r)\, e^{il\varphi}\ \hat{e}_r$, where l is the total topological charge, and E_0 is a constant. The intensity distribution at 15 nm above the HMM is shown in FIG. S4.

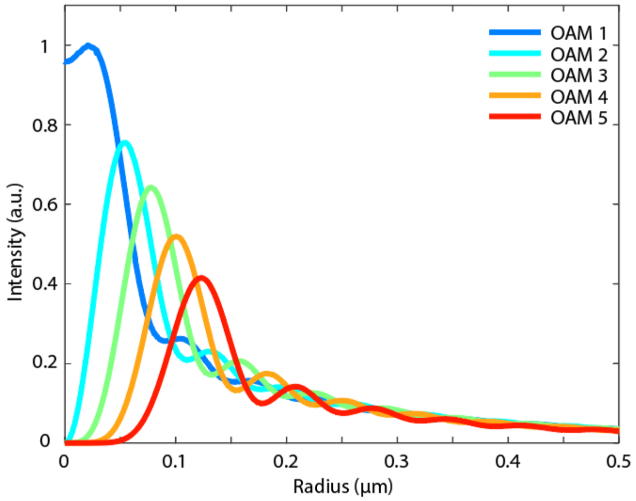

FIG. S4. Intensity distribution at 15 nm above the HMM for beams with topological charge 1–5.

With higher OAM, the focused spot size is also getting larger. Here we define the spot size using the diameter of the donut shape, which is set to be the position where the intensity drops to half of the maximum. The focused spot sizes for beams carrying OAM charge 1–5 are 120 nm, 170 nm, 220 nm, 270 nm, and 310 nm, respectively.

S7. Hypergrating Fabrication

The hypergrating fabrication process is shown in FIG. S5. The Fresnel gratings were fabricated by milling the designed concentric rings on a 50 nm thick Cr layer with a focused ion beam (FIB). The Fresnel gratings were filled with PMMA for planarization. Around a 250 nm PMMA layer was spin-coated on top of the Fresnel gratings, and the bulk area above the Cr surface was removed with reactive ion etching (RIE).

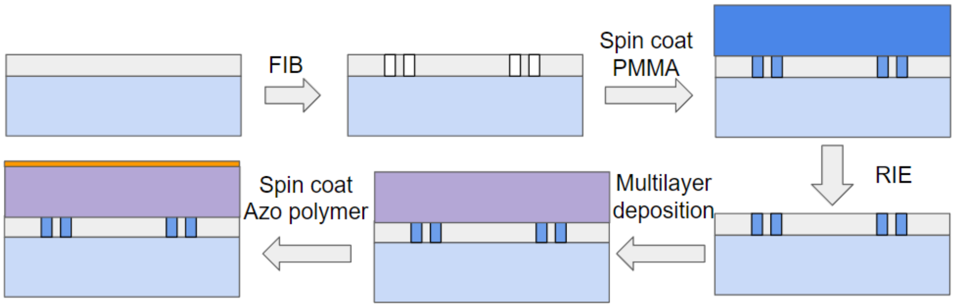

FIG. S5. Hypergrating fabrication procedures.

On top of the Fresnel grating, 20 alternating layers of 30 nm thick Ag and Ti_3O_5 were deposited using E-beam evaporation. The thicknesses of the layers were calibrated with ellipsometry measurements.

S8. Azo-Polymer Surface Topology Characteristics

Since the beam was confined within the hypergrating and only the evanescent wave was above the HMM, a near-field method was required to probe the focused beam. We used the surface relief pattern of the Azo-polymer to map out the area under illumination. A 40 nm thick layer of Azo-polymer was spin-coated on top of the hypergrating to probe the intensity distribution of the evanescent field. A circularly polarized 532 nm laser was focused on the back focal plane of the incident objective to create a plane wavefront at the input of the hypergrating sample, while the sample can be imaged with the same objective as is shown in FIG. S2. The hypergrating was placed under 216 W/cm^2 illumination for 5 minutes, and the surface topology of the Azo-polymer was measured with atomic force microscopy. The surface topology of the Azo-polymer before and after illumination is shown in FIG. S6 (a) and (b).

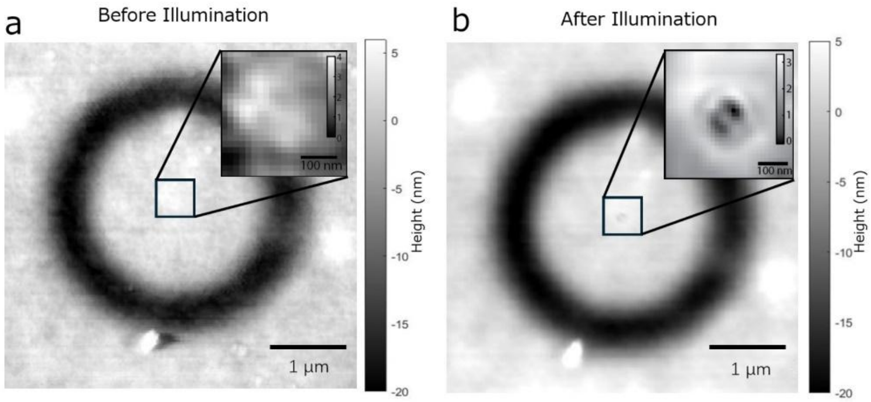

FIG. S6. Surface topology of the Azo-polymer before and after exposure. The colormap range used in (a) and (b) spans from the lowest point on the dark annulus to the highest point within the enclosed area.

Note that a donut-shaped valley was observed before and after illumination at the position of the Fresnel gratings and is about 20 nm deeper than the surroundings. The size of the valley stays the same before and after the illumination, as is shown in Fig. S6 (a) and (b). This valley resulted from the non-planarity of the hypergrating device, which can also be observed in the hypergrating cross-section plot (main paper, FIG. 2(b)). After exposure, an additional figure-8-shaped surface relief pattern becomes apparent at the focal point of the hypergrating. This pattern arises due to the illumination of the focused beam from the hypergrating.

S9. Surface Relief Pattern Formation in Azo Polymer

The surface relief pattern formed on the Azo-polymer after exposure originates from mass currents, which are influenced by both the light intensity and its gradient [3]. It is important to note that the valleys and peaks in the pattern do not directly correspond to the light intensity distribution. As an example, in Ref. [3], Ambrosio, A., et al. reported that the surface relief pattern forms a “double-arm spiral" structure under a vortex beam illumination, which bears some resemblance to the "figure 8" pattern observed in our work, though it differs in scale and height. And it has been suggested that such a pattern may appear when “an interference between the transverse optical field components E_x and E_y and the longitudinal component E_z is involved in the process”, which aligns with our predictions for the focused light field produced by the hypergrating. Since these surface relief patterns only form in illuminated areas, we can use the size of these patterns to estimate the size of the focused beam. Fig.S7 illustrates this estimation, where the red circle has a radius of 100 nm, indicating the focused beam's size.

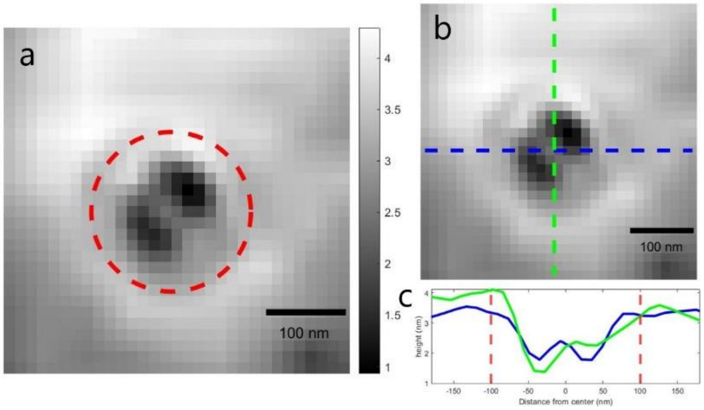

FIG. S7. Beam size estimation based on the surface relief pattern of Azo-polymer.

[1] Elser, J., et al., Nonlocal effects in effective-medium response of nanolayered metamaterials. Appl. Phys. Lett., 2007. 90(19): p. 1109.

[2] Singh, K., et al., Digital Stokes polarimetry and its application to structured light: tutorial. Journal of the Optical Society of America A-Optics Image Science and Vision, 2020. 37(11): p. C33-C44.

[3] Ambrosio, A., et al. Light-induced spiral mass transport in azo-polymer films under vortex-beam illumination. Nat. Comm., 2012. 3(1): p. 989.
